# Genetic polymorphism of *Plasmodium falciparum msp-1, msp-2* and *glurp* vaccine candidate genes in pre-artemisinin era clinical isolates from Lakhimpur district in Assam, Northeast India

**DOI:** 10.1099/acmi.0.000350

**Published:** 2022-04-25

**Authors:** Vinayagam Sathishkumar, Tulika Nirmolia, Dibya Ranjan Bhattacharyya, Saurav Jyoti Patgiri

**Affiliations:** ^1^​ ICMR-Regional Medical Research Centre, North East Region, Dibrugarh 786001, Assam, India

**Keywords:** *Plasmodium falciparum*, vaccines, drug resistance, Merozoite surface protein, India, glutamate-rich protein

## Abstract

**Background:**

Northeast India shares its international border with Southeast Asia and has a number of malaria endemic zones. Monitoring genetic diversity of malaria parasites is important in this area as drug resistance and increasing genetic diversity form a vicious cycle in which one favours the development of the other. This retrospective study was done to evaluate the genetic diversity patterns in *Plasmodium falciparum* strains circulating in North Lakhimpur area of Assam in the pre-artemisinin era and compare the findings with current diversity patterns.

**Methods:**

Genomic DNA extraction was done from archived blood spot samples collected in 2006 from malaria-positive cases in Lakhimpur district of Assam, Northeast India. Three antigenic markers of genetic diversity were studied – *msp-1* (block-2), *msp-2* (block-3) and the *glurp* RII region of *P. falciparum* using nested PCR.

**Results:**

Allelic diversity was examined in 71 isolates and high polymorphism was observed. In *msp-1,* eight genotypes were detected; K1 (single allele), MAD20 (six different alleles) and RO33 (single allele) allelic families were noted. Among *msp-2* genotypes, 22 distinct alleles were observed out of which FC27 had six alleles and IC/3D7 had 16 alleles. In RII region of *glurp,* nine genotypes were obtained. Expected heterozygosity (*H*_E_) values of the three antigenic markers were 0.72, 0.81 and 0.88, respectively. Multiplicity of infection (MOI) values noted were 1.28, 1.84 and 1.04 for *msp-1, msp-2* and *glurp,* respectively.

**Conclusion:**

Results suggest a high level of genetic diversity in *P. falciparum msp* (*block-2* of *msp-1* and *block-3 of msp-2*) and the *glurp* RII region in Northeast India in the pre-artemisinin era when chloroqunine was the primary drug used for uncomplicated *falciparum* malaria. Comparison with current studies have revealed that the genetic diversity in these genes is still high in this region, complicating malaria vaccine research.

## Introduction

Malaria is caused by the genus *Plasmodium,* five species of which are known to cause primary human infection. In tropical and subtropical regions, malaria has become a major health problem. The majority of mortality occurs in young children below 5 years of age residing in tropical regions. In 2020, the estimated global malaria case burden was 241 million with 627 000 deaths [1]. The WHO African region contributes 95 % of malaria cases whereas Southeast Asia contributes 2 % [1]. In humans, *Plasmodium falciparum* causes a severe form of malaria with high morbidity and mortality and there are reports of dissemination of drug-resistant strains from northeast India to other parts of the country [2].

A number of vaccines have been tried against the erythrocytic stage of the malaria parasite using different types of surface proteins like merozoite surface protein (MSP), apical membrane antigen-1 (AMA-1) and erythrocytic binding antigen-175 (EBA-175). The most abundant surface antigen in *P. falciparum* is MSP [3, 4]. *Pf*MSP-1/2/4/5/10 family consists of glycosyl phosphatidyl inositol (GPI) anchored proteins and *Pf*MSP-3/6/7/9 family members are soluble MSP proteins but they belong to other anchored GPI proteins [5]. MSP is amongst the forefront antigens for producing an effective vaccine against the blood stage of malaria parasites; these proteins are exposed to the immune system and they are present in the merozoites. GPI anchored MSPs play a vital role in the entire life cycle of the parasite and cannot be detached or knocked out. MSP is involved in binding of erythrocyte and also in complex formation with other MSP parasite proteins [6]. The most important function of MSP is invasion of mature erythrocytes via the sialic acid (SA) dependent and independent pathways [7]. *Msp-1*(block-2), *msp-2* (block-3) and *glurp* (RII-region) genes have highly polymorphic variations throughout the world and are expressed throughout the life cycle of *P. falciparum* [8, 9]. *Msp*-1 contains 17 blocks of which block-2 has K1, MAD20 and RO33 allelic families [10]. Block-3 of *msp*-2 contains two alleles viz. FC27 and IC/3D7 [11]. *Msp*-based vaccines are difficult to achieve because of the presence of several polymorphic genes and high allelic diversity [12]. The most successful pre-erythrocytic stage vaccine RTS,S/AS01 underwent multiple phases of clinical trials and has shown protection rates of only 39 % for malaria and 29 % for severe malaria [13]. Multiple clones of parasite infecting the host might be a major contributing factor for these low efficacy numbers [14]. Currently, the vaccine has been utilized in a pilot programme at Malawi in children aged up to 2 years [15].

In India, chloroquine (CQ)-resistant *P. falciparum* was first reported from Karbi-Anglong district in Assam in 1973 and in 1979, sulfadoxine/pyrimethamine (SP) resistance in *P. falciparum* was also reported in the same area [16, 17]. In late 1990s, SP-resistant *P. falciparum* was reported from Changlang, Arunachal Pradesh district (NMEP 1997). With widespread resistance, the anti-malarial drug policy was changed for Northeast India in 2008, heralding the beginning of artemisinin based combination therapies (ACT) [18]. It is possible that Northeast India may act as a gateway for the entry and dissemination of drug-resistant *P. falciparum* to the rest of India [19]. Increasing genetic diversity does not bode well for the possible future implementation and effectiveness of malaria vaccine candidates in this region. Selective antibiotic pressure and also changes of drug policy leads to a gradual increase in parasite diversity patterns with resistant genotypes [20]. Drug resistance and increasing genetic diversity form a vicious cycle in which one favours the development of the other. This retrospective study evaluated the genetic diversity patterns in parasite strains circulating in the North Lakhimpur area of Assam in the pre-artemisinin era to observe any differences with current diversity patterns. A nested PCR-based protocol was adopted to study the allelic variations in *P. falciparum glurp, msp-1* and *msp-2* antigens in isolates from Lakhimpur district, Assam.

## Methods

### Study site and population

The samples utilized in the present study were archived samples collected in 2006 from Nowboicha Primary Health Centre area, Lakhimpur, Assam [21]. It is situated approximately between longitude 94.1514˚ E and latitude 27.2064˚ N with an area of 2277 Km^2^ and population of 10,42,137 as per 2011 census data. This district receives high rainfall (annually 3215 mm) with an average temperature of 23.8 °C. The region also contains many tea gardens and dense forests. This region was selected as the study site since it is one of the malaria endemic district of Assam and shares its northern border with Arunachal Pradesh, which is again a malaria endemic state of Northeast India.

### Sample collection

Patients having primary malaria like clinical symptoms such as intermediate fever, chill, vomiting and body ache were screened by rapid diagnostic test (RDT) kit (Falcivax/Xephyr biomedical, India) for *P. falciparum* (HRP2) and *P. vivax* (LDH) followed by blood smear preparation for microscopy. Finger-prick blood samples from RDT positive, mono-infective, uncomplicated (as per WHO classification) *P. falciparum* cases were collected in Whatman filter paper (FTA card) after obtaining written consent from the participants. A total of 100 FTA card blood samples were collected, air-dried and preserved at −20 °C. The archived samples from 2006 were maintained in our department and 71 of these isolates were used for the current study. The remaining 29 FTA card blood samples could not be restored; they were spoiled and fragmented due to the storage problem in the freezer. The samples used in the current study were completely anonymised and no identifying features, names or other patient characteristics (age, sex, etc.) have been incorporated in the current study findings.

### DNA extraction from dry blood spots

Genomic DNA was isolated from each sample using six dried blood spots, 3 mm in diameter by Harris Uni-Core puncher. Between each sample, the Harris Uni-Core puncher was cleaned properly using 70 % ethanol followed by autoclaved double distilled water. DNA was isolated with the help of commercial kits (QIAamp DNA blood mini kit, Qiagen) and stored at −20 °C for further processing.

### PCR-based identification of malaria parasite

*Plasmodium* species identification was done using nested PCR targeting the 18S small subunit of ribosomal RNA gene (rRNA) specific for *Plasmodium* species as described earlier [22]. In the primary PCR, 20 µl reaction volume containing 1 µl of genomic DNA, 0.25 µM primer (IDT-integrated DNA technologies, Bangalore, India) concentration and 1X readymade Master Mix (Promega, Madison, WI, USA) was used. In the secondary PCR, a 2 µl template from the primary PCR was used in 20 µl reaction mixture containing 1X readymade PCR Master Mix (Promega) and 0.5 µM primer (IDT) concentration. Both primary and secondary PCR were done in a Biorad C1000 system. Initial denaturation was done for 5 min at 94 °C followed by 35 cycles of denaturation for 30 s at 94 °C. Annealing was done for 1 min at 55 °C, extension for 1 min at 72 °C followed by a final extension at 72 °C for 5 min. In the *Plasmodium* species-specific PCR, amplification bands of 120 bp indicate *P. vivax* and 205 bp indicates *P. falciparum*. Laboratory-adapted 3D7 and DD2 strains were used as positive controls.

### Genotyping of vaccine candidate antigens

Nested PCR was performed to amplify the *msp-1* (block 2), *msp-2* (block 3) and region 2 (RII) of *glurp* and their allelic variants. K1, MAD20 and RO33 families of *msp-1*, FC27 and 3D7/IC families of *msp-2* and region 2 (RII) of *glurp* were amplified using allele-specific primers as previously described [23]. For detection of allelic variants of *Pfmsp*-1, nPCR was undertaken according to the protocol by Soulama *et al.* [24]. For detection of *Pfmsp-1, Pfmsp-2* and *glurp* allelic variants*,* primary PCR was performed in a 20 µl reaction volume containing 1 µL of DNA, 0.125 mM (for *Pfmsp-*1), 0.25 mM (for *Pfmsp*-2 and *glurp*) of primer (IDT) and 1X readymade PCR Master Mix (Promega). Primary PCR cycling conditions for all three genes were: initial denaturation for 5 min at 95 °C followed by 25 cycles of denaturation for 1 min at 94 °C, annealing for 2 min at 58 °C and extension for 2 min at 72 °C. This was followed by a final extension at 75 °C for 5 min. Secondary PCR reactions were run on a Biorad C1000 Thermal cycler using 2 µl of primary PCR product as template, 0.25 mM primer (IDT) and 1X readymade PCR Master Mix (Promega). Secondary cycling conditions of PCR were as follows: one step denaturation for 5 min at 95 °C followed by 25 cycles of denaturation for 1 min at 94 °C, annealing at 61 °C for 2 min (for *Pfmsp*-1 and *Pfmsp*-2), 58 °C for 2 min (for *glurp*) and extension for 2 min at 72 °C. A final extension step was carried out at 75 °C for 5 min.

In allele-specific nested PCRs, the amplified DNA fragments were grouped according to their sizes: for *Pfmsp*-1 and *Pfmsp*-2 the alleles were considered identical if the fragment sizes were within 10 bp; a larger interval of 50 bp was considered for *glurp* as previously described [8, 25].

### Multiplicity of infection (MOI) and heterozygosity (*H*_E_)

To identify MOI obtained in *msp1, msp2* and *glurp* genes, PCR fragments were grouped as previously described [25]. Monoclonal infection was identified by a single fragment in the loci whereas multiple PCR fragments in the loci indicated a polyclonal infection. The ratio of alleles derived for each gene to the PCR positive samples for the same gene was defined as MOI [26]. Expected heterozygosity (*H*_E_) was estimated as described previously elsewhere [8].

The distribution pattern of *Plasmodium* species varies among the eight Northeastern states of India. In Assam, predominant species is the *P. falciparum.* Therefore, the study was restricted to this *Plasmodium* species only. Apart from *P. falciparum* and *P. vivax,* other human malaria parasites reported from Assam are *P. ovale* and *P. malariae*. The first and only *P. ovale* case reported was from Jorhat district of Assam [27]. One *P. malariae* case was reported from Assam and from two other states of Northeast India, viz: Arunachal Pradesh and Tripura, the same species was also reported [28–30]. So far, no study has been carried out in this region of India for other species of *Plasmodium* based on their genetic diversity pattern. Although *P. vivax* is co-endemic in northeastern states of India, it still remains neglected.

## Results

### Genotyping of *msp-1*, *msp-2* and *glurp* antigens

Dried blood spots collected from 71 *P. falciparum* positive cases were included in this study. All these samples were mono-infection cases and were positive for *P. falciparum* by genus specific PCR. Out of these samples, *Pfmsp1*, *Pfmsp2* and *Pfglurp* genes could be successfully amplified individually in 64 (90.14 %), 63 (88.73 %) and 65 (91.54 %) samples, respectively. A total of eight different allelic variants of *msp-1*, 22 allelic variants of *msp-2* and eight allelic variants of *glurp* were detected. The MOI detected for *msp-1*, *msp*-2 and *glurp* were 1.28, 1.84 and 1.04, respectively.

**Fig. 1.**
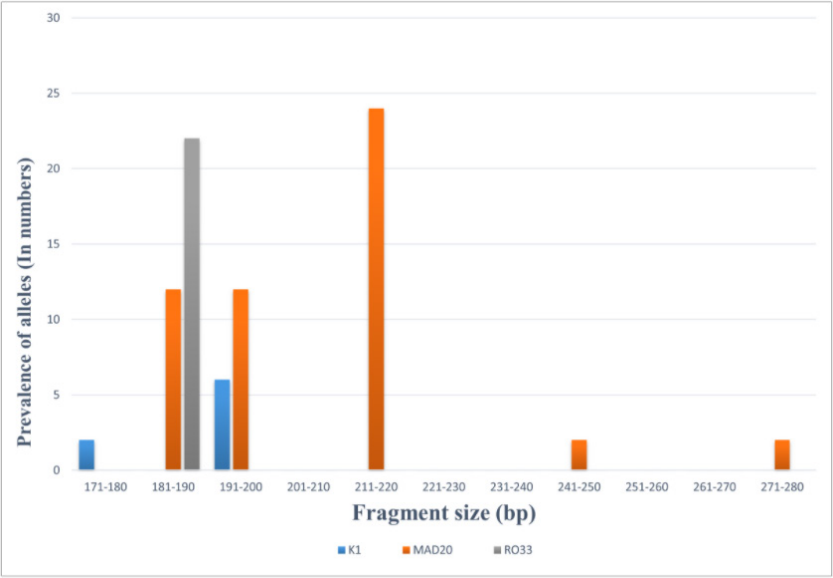
Allele frequencies of *P. falciparum msp*-1 detected by nPCR from archived (2006) samples collected in Lakhimpur, Assam.

In *Pfmsp-1* locus, the dominant allelic family was MAD20 (68.75 %, *n*=44) with six different allele fragments in the range of 180–280 bp; the 220 bp fragment size was predominant (40.91 %, *n*=18). Among the nine allelic families of MAD20, the following allelic combinations were observed: 180 and 220 bp (*n*=1), 190 and 200 bp (*n*=1), 190 and 220 bp (*n*=4), 200 and 250 bp (*n*=2) and 220 and 280 bp (*n*=2). Both K1 (9.38 %, *n*=6) and RO33 (34.38 %, *n*=22) allelic families showed only one allelic variant of 200 and 190 bp, respectively. The frequency of monoclonal infection and polyclonal infection was 87.5 % (*n*=56) and 12.5 % (*n*=8), respectively. The detected MOI value and *H*_E_ were 1.28 and 0.81 (Table 1). PCR-based allele frequencies of *Pfmsp-1* are shown in Fig. 1.

**Table 1.** *Pfmsp-1* (block II region) allelic variants of *P. falciparum* detected by nPCR from archived (2006) samples collected in Lakhimpur, Assam

Genotypes	Frequency (%)	Allele size (bp)	No. of alleles	Monoclonal infection	Polyclonal infection	MOI	*H*_E_
	(*n*=64)					(*n*=64)	
**K1**	6 (9.3 %)	200	1	87.50 %	12.50 %	1.28	0.81
**MAD20**	44 (68.7 %)	180–280	6				
**RO33**	22 (34 %)	190	1				

bp – base pair.

**Fig. 2.**
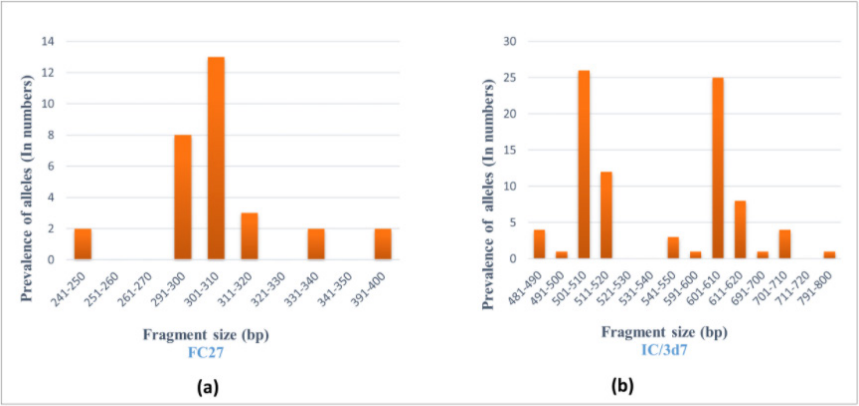
Allele frequencies of *P. falciparum msp*-2 detected by nPCR from archived (2006) samples collected in Lakhimpur, Assam: (a) frequency of FC27; (b) frequency of IC/3D7.

In *Pfmsp-2* locus, the dominant and highly polymorphic allelic family was IC/3d7 (80.95 %, *n*=51) with 16 different fragments (490–700 bp) and the predominant fragment size was 510 bp (7.84 %, *n*=4). In the IC/3d7 allelic family, among the 16 alleles, multiple bands were observed in nine allele families. Out of these, the 510 and 610 bp combination was predominant (39.22 %, *n*=20), followed by 520 and 620 bp (*n*=5), 520 and 610 bp (*n*=2), 710 and 620 bp (*n*=2), 490 and 510 bp (*n*=2), 500 and 610 bp (*n*=1), 610 and 710 bp (*n*=1), 700 and 810 bp (*n*=1), 490 and 520 bp (*n*=1). FC27 allelic family (47.62 %, *n*=30) showed six different allele fragments (250–400 bp) and 310 bp (43.33 %, *n*=13) fragment size was predominant. The frequency of monoclonal infection and polyclonal infection were 71.43 % (*n*=45) and 28.57 % (*n*=18), respectively. The detected MOI value for *Pfmsp-2* was 1.84 and expected heterozygosity was 0.88 (Table 2). PCR-based allele frequencies of *Pfmsp-2* are shown in Fig. 2.

**Table 2.** *Pfmsp-2* block III allelic variants of *P. falciparum* detected by nPCR from archived (2006) samples collected in Lakhimpur, Assam

Genotypes	Frequency (%)	Allele size (bp)	No. of alleles	Monoclonal infection	Polyclonal infection	MOI	*H*_E_
	(*n*=63)					(*n*=63)	
**FC27**	30 (47.6 %)	250–400	6	71.42 %	26.98 %	1.84	0.88
**IC/3d7**	51 (80.9 %)	490–700	16	(*n*=45)	(*n*=18)		

bp – base pair; *H*_E_ – expected heterozygosity.

**Fig. 3.**
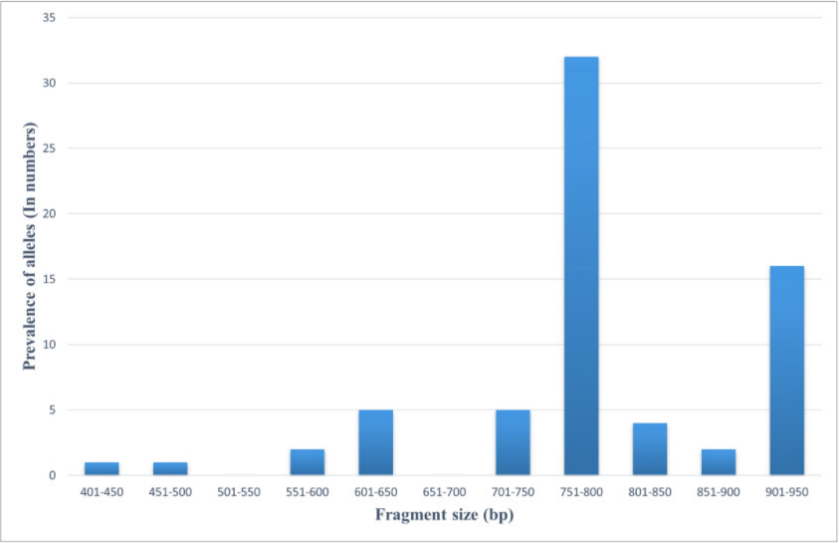
Allele frequencies of *P. falciparum glurp* detected by nPCR from archived (2006) samples collected in Lakhimpur, Assam.

In the RII region of *Pfglurp*, nine different allelic fragments (genotype I–IX) were observed. The most commonly detected allelic variant was the 800 bp fragment, which was denoted as genotype VI (47 %, *n*=32) followed by 900–950 bp genotype IX (23.52 %, *n*=16). In both the genotypes IV and V, the frequency of allelic variants was 7.35 % (*n*=5). Genotype VII was detected in four samples (5.88 %) and genotype I and II were determined in only one sample each (1.47 %). Among the nine different allele fragments, multiple infections were found in three cases (Table 3). The frequencies of both monoclonal and polyclonal infections were 95.38 % (*n*=62) and 4.61 % (*n*=3), respectively. The detected MOI value and expected heterozygosity were 1.04 and 0.72, respectively (Table 3). PCR based allele frequencies of *Pfglurp* are shown in Fig. 3. A comparison of the m.o.i. over the years in India and other countries from South-East Asia is shown in Table 4.

**Table 3.** *Pfglurp* RII region allelic variants of *P. falciparum* detected by nPCR from archived (2006) samples collected in Lakhimpur, Assam

Genotypes	Frequency (%)	Allele size	Monoclonal infection	Polyclonal infection	MOI	*H*_E_
	(*n*=65)	(50 bp bin)			(*n*=65)	
**I**	1 (1.47 %)	400–450	95.38 % (*n*=62)	4.61 % (*n*=3)	1.04	0.72
**II**	1 (1.47 %)	451–500				
**III**	2 (2.94 %)	551–600				
**IV**	5 (7.35 %)	601–650				
**V**	5 (7.35 %)	701–750				
**VI**	32 (47 %)	751–800				
**VII**	4 (5.88 %)	801–850				
**VIII**	2 (2.94 %)	851–900				
**IX**	16 (23.52 %)	901–950				

bp – base pair; *H*_E_ – expected heterozygosity.

**Table 4.** Comparison of MOI in India and other South-East Asian countries over the years

Sl. No.	Reference	Study period	Study area	MOI			*H*_E_		
				*msp-1*	*msp-2*	*glurp*	*msp-1*	*msp-2*	*glurp*
**1**	Present study	2005–2006	Lakhimpur, Assam, NE India	1.28	1.84	1.04	0.81	0.88	0.72
**2**	Joshi *et al.* 2007 [32]	na	Karbi Anglong, Assam	2.1	1.2	–	–	–	–
			Kamrup, Assam	1.4	1.1	–	–	–	–
			Keonjhar, Orissa	1.1	1.3	–	–	–	–
			Sundergarh, Orissa	1.2	1.6	–	–	–	–
			Darjeeling, West Bengal	1.1	1	–	–	–	–
**3**	Bharti *et al.* 2012 [42]	2005	Madhya Pradesh, Central India	1.27	–	–	0.94	–	–
		2009		1.34	–	–	0.91	–	–
**4**	Kumar *et al.* 2014 [61]	2005 2011	Assam, NE India	–	–	1.21 1.12	– –	– –	0.87 0.85
**5**	Akter *et al.* 2012 [70]	2004, 2005 and 2008	Bangladesh	2.7	–	1.2	–	–	–
**6**	Mohd Abd Razak *et al.* 2016 [73]	2008–2009	Kalabakan, East Malaysia	1.65	1.2	–	0.17	0.37	0.70
		2011 and 2014	Kota Marudu, East Malaysia	1.05	1.05	–	0.24	0.25	0.69
**7**	Patel *et al.* 2017 [20]	2013–2015	Chhattisgarh, Central India	1.67	1.28	–	–	–	–
**8**	Patgiri *et al.* 2019 [51]	2015	Tripura, NE India	1.56	1.31	1.06	0.89	0.81	0.85
**9**	Hussain *et al.* 2011 [35]	2008	Jharkhand, India	1.38	1.39	–	–	–	–
**10**	Atroosh *et al.* 2011 [74]	na	Malaysia	1.37	1.2	–	0.57	0.55	–
**11**	Congpuong *et al.* 2014 [71]	2009, 2012–2013	Thai-Myanmar Border	2.15	2.93	1.26	–	–	–
**12**	Soe *et al.* 2017 [72]	2009–2010	Myanmar	1.94	–	–	–	–	–
**13**	Yuan *et al.* 2012 [75]	2007 2008 2009 2010	China-Myanmar Border	1.34	–	–	–	–	–
				1.22	–	–	–	–	–
				1.62	–	–	–	–	–
				1.46	–	–	–	–	–

*H*_E_ – expected heterozygosity.

## Discussion

The present study describes the genetic diversity patterns of *P. falciparum* potential vaccine candidate antigenic genes *msp-1, msp-2* and *glurp* in archived samples collected in 2006 from a malaria endemic region of Assam, Northeast India. These *P. falciparum* isolates belong to the pre-artemisinin era when chloroquine was the mainstay of treatment for *falciparum* malaria. The genetic diversity patterns in terms of allelic variants, MOI and *H*_E_ observed in that period, when compared to those observed in published current and historical studies conducted in geographically similar areas, have the potential to reveal temporal variation in diversity patterns. This information adds to the existing knowledge of *P. falciparum* genetic diversity in field-collected samples and can provide important leads for vaccine research.

Various parts of Northeast India are highly malaria endemic where persistent malaria transmission is reported [31]. Numerous studies have reported that the allele frequency of each gene is associated with the degree of endemicity. In high endemicity areas such as Asia and Africa, a greater number of alleles were reported whereas in low endemicity areas, the number of alleles detected was lower [32–36]. In our study, all the reported allelic families of *msp-1* (K1, RO33 and MAD20), *msp-2* (FC27 and IC/3d7) and RII region of *glurp* were observed.

MSP-1 has a molecular weight of 195 KDa and is present in both merozoites and mature schizonts; *msp-1* is dimorphic and contains three genotypes (K1, MAD20, and RO33) and sequence analysis has revealed that it consists of 5300 bases which code for more than 1700 amino acids [37]. In our study, we have observed that the 220 bp MAD20 (*n*=44) allele of *msp*-1 is predominant followed by RO33 (*n*=22) and K1 (*n*=6) which is in contrast to reports from North-West Colombia [38], Indonesia [39], Ethiopia [9], Central India [20] and previous studies performed in different regions of Northeast India [32, 40–42]. Myanmar reported MAD20 as dominant allele type [43, 44]. Tripura reported predominance of *msp-1 K1* (38) allele and Arunachal Pradesh reported that the *msp1-RO33* allele was dominant and both these findings are in contrast to our study findings [19]. A recent study from Africa also demonstrated that the distribution of *msp-1* alleles had changed in high transmission seasons and one dominant allele was replaced by another allele type [45].

Our samples were collected before ACT drug policy was implemented in Northeast India when CQ was the first-line malaria treatment and our results are similar to that observed in North-West Colombia (1997) where CQ was the first line therapy for malaria [38]. The change in anti-malarial drug policy in 2008 from chloroquine to ACT might have had some impact on the genetic diversity patterns observed. It was documented that change in local anti-malarial drug policies could cause dynamic changes in genetic diversity of a parasite population [46]. In a study carried out in Africa, it was observed that parasite diversity was progressively decreased after the introduction of ACT, since the disease transmission intensity was reduced [47]. A study from Bioko island also reported that after implementing several anti-malarial drug policies, genetic variations of msp-1 and msp-2 alleles were higher than before [48].

MSP-2 is a small molecular protein (45–52 KDa) located in chromosome-2 and is expressed in merozoites. It is mainly associated with high anti-immunogenicity and increased disease morbidity [49]. The current study showed that the IC/3d7 allele with 16 allelic types was dominant over FC27, which corroborates with previous studies performed in Myanmar [43, 44], Cameroon [50], Central India [20] and Tripura, Northeast India [51]. In our study, nine types of *msp*-2 allelic combinations were observed; the 510 bp with 610 bp combination was dominant. These results are in contrast with a previous Indian study carried out in Orissa, Madhya Pradesh and Rajasthan [52]. In an earlier study carried out in India with a very small number of *P. falciparum* isolates, only the FC-27 allele type was reported [53, 54]. From many parts of the world, viz. Senegal [55], Gambia [56], Colombia [57], Honduras [34] and Papua New Guinea [58], the FC-27 family has been reported as the predominant allele type over IC/3d7. As in *msp-1* alleles, this discrepancy could be due to the pattern of disease transmission intensity, change in drug policy, anti-malarial drug pressure and higher level of intragenic recombination in a parasite population [44, 45].

GLURP (glutamate-rich protein) is exo-antigenic in nature and has a molecular weight of 220 KDa. *Pf*GLURP antigen induces protective immunity and thereby has a vital role in malaria vaccine development [59, 60]. In Northeast India, only a few studies have been reported on polymorphism of *Pfglurp* RII region. The first study in Assam, Northeast India reported ten different types of *glurp* alleles based on their base pair sizes [61]. Similarly, in Nigeria, 12 alleles of *glurp* were reported before and after ACT implementation [62]. Later, nine allele types of *glurp* were reported from Northeast Indian states of Arunachal Pradesh [40] and Tripura [51]. Our study found nine different types of *glurp* alleles in Assam, which is similar to previous reports from Northeast India. In low endemic areas like Central South America [63], Honduras [34], Colombia [64] and Brazil [65], the frequency of *glurp* allele has been reported to range from two to four; while in endemic regions like India [66], Thailand [67] and Sudan [68], the *glurp* allele frequency is higher.

MOI and *H*_E_ are indirect measures of parasite genetic diversity in a particular region. The degree of transmission intensity usually regulates the rate of the MOI but the relationship of parasite prevalence and degree of endemicity with MOI is still inconclusive [69]. In this study, the detected MOI for the polymorphic genes *msp-1, msp-2* and *glurp* were found to range from 1.04 to 1.84. MOI of the polymorphic genes was similar to those reported earlier from Indian studies [20, 32, 51, 61]. Considering the differences in pre-artemisinin era vs. artemisinin era MOI values (Table 4), it can be seen that the MOI values for *Pfmsp-1* in the current study were marginally lower than those observed in Chhattisgarh [20] and Tripura [51]. Some areas like Bangladesh and Thai-Myanmar border are more endemic for malaria and have reported higher *Pfmsp-1* MOI values than Indian studies [70–72]. *Pfmsp-2* MOI values observed in the present study were, however, higher as compared to most Indian studies [20, 32, 35, 51]; but lower than that observed in the Thai-Myanmar border area [71]. *Pfglurp* MOI values did not show much variation in the pre-artemisinin vs. artemisinin era studies from Southeast Asia including India [51, 61, 70, 71]. Heterozygosity (*H*_E_) values observed in our study for *Pfmsp-1* and *Pfmsp-2* were slightly higher than observed in Malaysia [73, 74] but comparable to Indian studies [42, 51] whereas, for *Pfglurp,* it was slightly lower than those reported earlier from Tripura and Assam, Northeast India [51, 61]. Slight changes in MOI and *H*_E_ values over the years may be due to differences in geographical locations, sampling intervals and subsequent changes in the malaria drug policy in Northeast India.

## Conclusion

The present study evaluated the antigenic diversity of *P. falciparum* clinical isolates from 2006 in Assam, Northeast India before the implementation of ACT. While the malaria burden has reduced in the study area and entire Northeast India drastically over the years, a similar trend in reduction of *P. falciparum* genetic diversity parameters was not observed in comparison with newer studies. Information from wide-scale studies on genetic diversity of the malaria parasite may help in a better understanding of the population dynamics of the parasite and how this contributes towards the development of an efficient vaccine for elimination of malaria over time.
